# Identification and Validation of Signature Genes in Invasiveness-Associated Modules of Nonfunctioning Pituitary Adenomas

**DOI:** 10.3390/biomedicines14020484

**Published:** 2026-02-23

**Authors:** Xin Ma, Hongyu Wu, Yu Zhang, Zhijun Yang, Pinan Liu

**Affiliations:** 1 Department of Neurosurgery, Beijing Tiantan Hospital, Capital Medical University, Beijing 100070, China; 2 Department of Neural Reconstruction, Beijing Neurosurgery Institute, Capital Medical University, Beijing 100070, China

**Keywords:** NFPA, invasiveness, biomarker, KIFC3, multi-omics integration

## Abstract

**Background:** Invasive non-functional pituitary adenomas (NFPAs) are associated with high recurrence and unfavorable clinical outcomes, yet their underlying molecular mechanisms remain incompletely understood. This study aimed to identify robust biomarkers of invasiveness by integrating transcriptional networks, machine learning, and epigenetic regulation. **Methods:** RNA sequencing was performed on 32 NFPA samples (15 invasive, 17 non-invasive). Weighted gene co-expression network analysis (WGCNA) was used to identify invasiveness-associated modules, which were validated in public datasets (GSE169498, GSE51618). Candidate genes were prioritized using machine learning, and their epigenetic regulation was studied using DNA methylation datasets (GSE207937, GSE115783). **Results:** We identified a five-gene signature associated with invasiveness (KIFC3, PNMA3, ARHGAP18, LRRC10B, and KCNC4). All five genes were consistently downregulated in invasive NFPAs (all *p* < 0.01) and were enriched in oxidative phosphorylation and neuroactive ligand–receptor interaction pathways. A machine learning validation approach (Random Forest followed by forward stepwise logistic regression) showed strong discriminative performance for this signature (mean AUC = 0.919). DNA methylation analyses indicated no robust differences at the genome-wide level or across promoter regions of the core genes; nevertheless, several locus-specific CpG sites (e.g., near KIFC3) showed suggestive methylation changes. **Conclusions:** Using an integrative multi-omics framework, we identified a novel five-gene signature associated with NFPA invasiveness. The coordinated downregulation of these genes may reflect alterations in cellular energy metabolism and microenvironmental signaling. Although the signature demonstrated promising diagnostic potential, its transcriptional repression is unlikely to be primarily explained by DNA methylation. These findings provide candidate markers and mechanistic hypotheses for understanding invasive NFPA and developing risk-stratification tools.

## 1. Introduction

Pituitary adenomas (PAs) are among the most common intracranial benign tumors, with an estimated annual incidence of 3.9–7.4 cases per 100,000 individuals and a prevalence of 76–116 cases per 100,000 in the general population [1,2]. Based on hormone secretion, PAs are classified into functioning pituitary adenomas (FPAs) and nonfunctioning pituitary adenomas (NFPAs) [3]. FPAs hypersecrete pituitary hormones (e.g., prolactin, growth hormone) and cause characteristic endocrine syndromes; For example, prolactinomas can lead to amenorrhea–galactorrhea, whereas growth hormone-secreting adenomas lead to acromegaly. In contrast, NFPAs typically lack overt hormonal hypersecretion and may remain clinically silent in their early stages, often being detected after tumor enlargement produces mass effects [4,5].

The biological behavior of NFPAs is heterogeneous, with approximately 35% of cases exhibiting invasive growth [6]. The diagnosis of invasive NFPAs typically integrates radiological, pathological, and intraoperative evidence, including imaging criteria (Knosp grade ≥ 3), proliferative markers (Ki-67 index ≥ 3%, aberrant p53 expression), and surgical confirmation of cavernous sinus invasion [7,8]. Because gross total resection is difficult to achieve and responses to conventional radiotherapy may be limited, these invasive tumors show a high 5-year recurrence rate (40–60%) and are associated with substantial morbidity (e.g., neurological deficits and hypopituitarism) and, in severe cases, mortality. Despite this clinical burden, the molecular determinants of invasive NFPA behavior remain incompletely defined. Prior work implicates processes such as extracellular matrix remodeling [9,10]; however, traditional single-gene strategies are often insufficient to capture the polygenic and network-level mechanisms that underlie invasiveness, limiting the development of robust prognostic biomarkers and therapeutic targets.

Therefore, we integrated weighted gene co-expression network analysis (WGCNA) with machine-learning approaches to construct an invasiveness-associated co-expression network in an internal cohort of 32 NFPA samples (15 invasive vs. 17 non-invasive) and to validate the resulting modules and candidate genes in independent public databases. Our aim is to identify core regulatory modules linked to invasiveness and characterize their potential functional implications, thereby suggesting a rationale for developing multigene biomarker-based risk stratification and for prioritizing candidates for future therapeutic exploration.

## 2. Materials and Methods

### 2.1. Sample Collection and Grouping

This study employed an integrative analytical strategy, incorporating an internally collected cohort and independent cohorts from public databases. The internal cohort comprised 32 fresh-frozen nonfunctioning pituitary adenomas (NFPAs) tissue samples obtained from the surgical specimen repository of the Department of Neurosurgery, Beijing Tiantan Hospital, Capital Medical University, from patients who underwent surgery between 2024 and 2025. All tissues were snap-frozen in liquid nitrogen within 30 min of surgical resection and subsequently stored at −80 °C until nucleic acid extraction.

The sample size of the internal cohort was primarily determined by the practical availability of surgical specimens meeting predefined clinicopathological criteria. To quantify the sensitivity of this design, we performed a post hoc power (sensitivity) analysis using G*Power (v3.1.9.7). Assuming a two-sided two-sample *t* test, α = 0.05, and 1 − β = 0.80, and using the observed group sizes (non-invasive, *n* = 17; invasive, *n* = 15), the minimum detectable standardized effect size was Cohen’s d = 1.03 (Figure S1). Accordingly, the discovery cohort was primarily powered to detect genes with relatively large expression differences between invasive and non-invasive tumors.

Tumor invasiveness was comprehensively determined using an integrated multimodal assessment based on radiological, intraoperative, and pathological evidence. The primary criterion was the Knosp grade based on preoperative magnetic resonance imaging (MRI): Knosp grades 3–4 were classified as invasive (*n* = 15), whereas grades 0–2 were classified as non-invasive (*n* = 17). Ancillary supportive criteria included: (1) intraoperative documentation of invasion into adjacent structures such as the cavernous sinus; and (2) proliferative markers in postoperative pathology (e.g., Ki-67 index ≥ 3%). Final group assignments were determined by consensus after independent review of imaging and clinical records by at least two senior neurosurgeons, who were blinded to the molecular results.

### 2.2. Inclusion and Exclusion Criteria

Inclusion Criteria:

(1) Age between 18 and 75 years.
(2) Histopathologically confirmed diagnosis of nonfunctioning pituitary adenoma (NFPA).
(3) No prior history of radiotherapy or targeted therapy before surgery.
(4) Complete pre-operative and post-operative imaging data and clinical follow-up data (≥6 months).

Exclusion Criteria:

(1) Coexistence of other endocrine tumors (e.g., pituitary carcinoma, metastatic tumors).
(2) History of prior medical treatment for the tumor.
(3) Pregnancy or severe physical/psychiatric comorbidities incompatible with surgery and follow-up.

### 2.3. Ethical Review and Informed Consent

This study was approved by the Ethics Committee of Beijing Tiantan Hospital and was conducted in accordance with the Declaration of Helsinki. Written informed consent was obtained from all participants before surgery for the use of their biological specimens in research.

### 2.4. RNA Sequencing and Transcriptomic Data Analysis

#### 2.4.1. RNA Extraction and Library Construction

Total RNA was extracted from frozen tissues, and subsequent cDNA library preparation was completed by BGI Genomics Co., Ltd. (Shenzhen, China).

#### 2.4.2. Sequencing Data Preprocessing and Normalization

Raw sequencing data (FASTQ) were initially quality-assessed using FastQC v0.12.1, and adapter sequences and low-quality reads were removed using Trimmomatic version 0.27. Clean reads were aligned to the human reference genome (GRCh38.p13) with HISAT2 2.1.0. Gene-level expression was performed with StringTie 2.1.7 and summarized as transcripts per million (TPM). Potential batch effects were adjusted using ComBat version 1.9 (sva R package), and the corrected TPM matrix was transformed as log_2_(TPM + 1) for downstream analysis.

#### 2.4.3. Differential Expression Gene Analysis

Differential expression analysis was performed using the limma R package 3.30.7. The invasive group (*n* = 15) was compared with the non-invasive group (*n* = 17) using the batch-corrected log_2_(TPM + 1) values. Differentially expressed genes (DEGs) were defined using the following criteria: |log_2_FC| > 2.55 (corresponding to the 95th percentile of |log_2_FC| across all genes) and FDR-adjusted *p* < 0.01.

#### 2.4.4. Weighted Gene Co-Expression Network Analysis

A weighted gene co-expression network was constructed using the WGCNA R package(v1.73). Genes with very low expression (TPM < 1 in ≥80% of samples) and low variability (standard deviation < 0.5 across samples) were filtered out. Sample clustering based on Euclidean distance did not identify obvious outliers. A soft-thresholding power (β) of 14 was chosen to approximate scale-free topology (fit index R^2^ > 0.9). A signed topological overlap matrix (TOM) was then computed, and gene modules were detected using the dynamic tree cut algorithm (parameters: deepSplit = 2, minModuleSize = 60). Modules with highly correlated eigengenes (correlation > 0.75) were merged, yielding nine final modules. Module-trait associations were evaluated using the Pearson correlation coefficient between module eigengenes and invasiveness status (binary variable), with *p* values adjusted using the Benjamini–Hochberg method.

#### 2.4.5. Hub Gene Screening Within the Core Module

Hub genes in the module most significantly associated with invasiveness were identified using three criteria: (1) intramodular connectivity, retaining genes in the top 10% of kWithin; (2) module membership, retaining genes with KME ≥ 0.8; and (3) gene significance, retaining genes with GS ≥ 0.2 for invasiveness. Genes meeting all three criteria were defined as hub genes for that module and were then intersected with the DEGs (Section 2.4.3) to obtain a high-confidence candidate gene set.

#### 2.4.6. Cross-Dataset Integration and Machine Learning Validation

For independent validation, GSE169498 (*n* = 73) and GSE51618 (*n* = 7) were downloaded from the GEO database. They underwent the same standardization and ComBat batch-correction pipeline as the internal cohort, and analyses were restricted to the intersecting gene set. Machine learning analysis was trained on the batch-corrected expression matrix (*n* = 112). Using the high-confidence candidate gene set as input features, we systematically compared 113 algorithms (including Random Forest, Support Vector Machine, and XGBoost 3.1.2) on the internal cohort (*n* = 32). To mitigate the impact of imbalanced validation sample sizes, model performance was assessed using two metrics: (1) the mean area under the receiver operating characteristic curve (AUC) across validation samples and (2) the sample-size-weighted AUC. The Random Forest followed by forward stepwise logistic regression (RF + Stepglm[forward]) showed the best overall performance. This model was then used to identify key feature genes, which were further evaluated using univariate ROC analyses. The top five genes, ranked by mean AUC, were selected to form the core signature gene set.

#### 2.4.7. Functional Enrichment and Interaction Network Analysis

Functional annotation was performed for the screened gene sets. Gene Ontology (GO, covering biological process, cellular component, and molecular function) and Kyoto Encyclopedia of Genes and Genomes (KEGG) enrichment analyses were conducted using the clusterProfiler R package. Statistical significance was defined at nominal *p <* 0.01 and FDR-adjusted *p* < 0.05. GeneMANIA version 2.3.6 was used to construct protein-protein interaction and functional association networks for the core signature genes. In addition, gene set enrichment analysis (GSEA) was performed based on the co-expression pattern of the core gene set using the clusterProfiler R package (version 4.14.6).

### 2.5. DNA Methylation Data Analysis

#### 2.5.1. Data Acquisition and Preprocessing

DNA methylation data were obtained from the public datasets GSE207937 and GSE115783. Raw IDAT files were processed using the minfi R package 1.36.0. Quality control steps included removal of probes with detection *p*-value > 0.01, cross-reactive probes, and probes located on sex chromosomes. Methylation intensities were converted to β-values (0–1), representing the proportion of methylation. Between-array normalization was performed using the beta-mixture quantile (BMIQ) method.

#### 2.5.2. Differential Methylation Analysis

Differentially methylated positions (DMPs) were identified using the champ.DMP 2.8.9 (ChAMP R package), which applies an empirical Bayes moderated linear model to compare the invasive (Knosp 3–4) and non-invasive (Knosp 0–2) groups. Significant DMPs were defined as β-value (|Δβ|) > 0.10 and *p*-value < 0.05. DMPs were annotated using the IlluminaHumanMethylationEPICanno.ilm10b4.hg19 annotation package.

#### 2.5.3. Targeted Methylation Analysis of Core Genes

**Figure 1:**
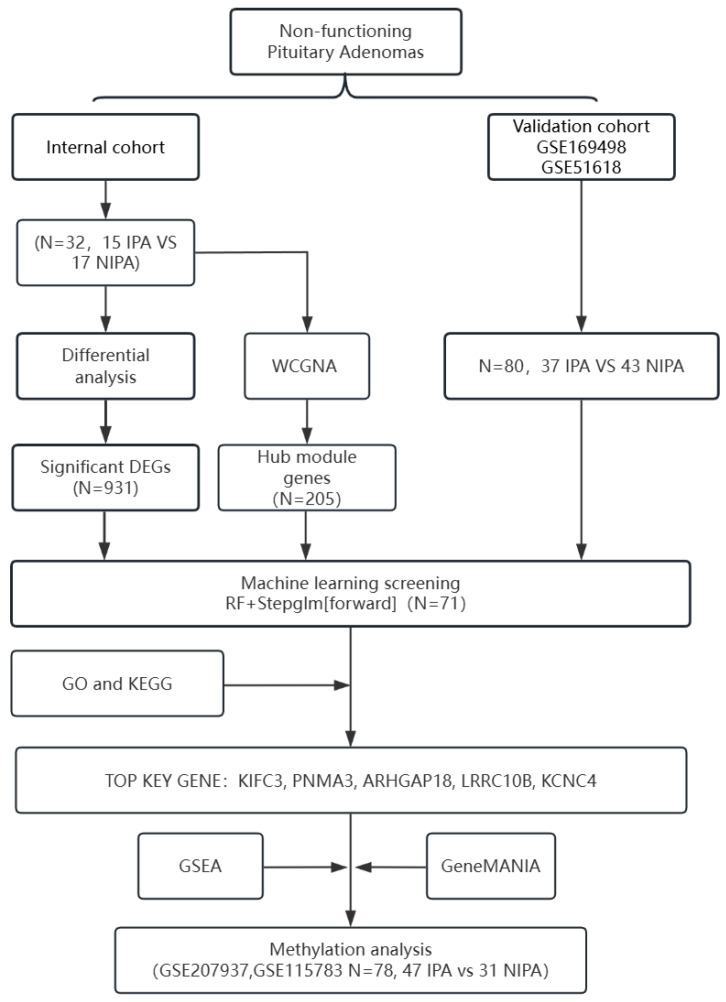
Schematic illustration of the analytical pipeline for identifying and validating signature genes associated with invasiveness in non-functioning pituitary adenomas (NFPAs). This diagram summarizes the entire workflow of the study, encompassing sample collection, processing of multi-omics data (transcriptome and methylation), screening of core modules and genes, cross-dataset machine learning validation, and functional analyses.

For the five core signature genes (KIFC3, PNMA3, ARHGAP18, LRRC10B, KCNC4) identified from the transcriptomic analysis, all CpG sites within their promoter regions were extracted. Promoters were defined as the transcription start site (TSS) ± 1500 bp. For each gene, the mean promoter (average β value across CpG sites) was calculated and compared between invasive and non-invasive groups. The overall design and analytical workflow of this study are summarized in Figure 1.

## 3. Results

### 3.1. Transcriptomic Landscape of Invasive vs. Non-Invasive Pituitary Adenomas

**Figure 2:**
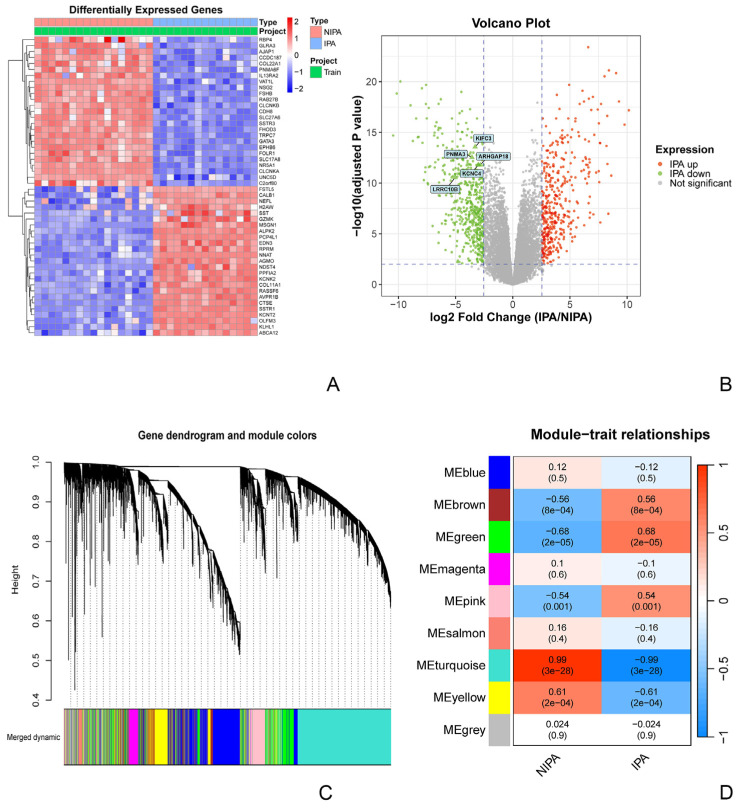
Global transcriptomic alterations and identification of an invasiveness-associated co-expression module in NFPAs. (**A**) Unsupervised hierarchical clustering of the top 50 most significantly differentially expressed genes (DEGs) clearly segregates invasive (*n* = 15) from non-invasive (*n* = 17) samples. (**B**) Volcano plot of all DEGs (|log_2_FC| > 2.55, FDR < 0.01). Upregulated and downregulated genes are shown in red and green, respectively. The five core signature genes (KIFC3, PNMA3, ARHGAP18, LRRC10B, KCNC4) are explicitly labeled. (**C**) Cluster dendrogram of genes obtained from WGCNA, with colors indicating the nine identified co-expression modules. (**D**) Heatmap depicting module-trait correlations. The turquoise module demonstrates the strongest positive correlation with the invasive phenotype (Pearson r = 0.99, *p* = 3 × 10^−28^).

To delineate transcriptomic alterations associated with invasiveness in nonfunctioning pituitary adenomas (NFPAs), we first performed differential expression analysis on our internal cohort (15 invasive vs. 17 non-invasive samples). This analysis identified 931 significantly differentially expressed genes (DEGs), comprising 414 upregulated and 517 downregulated genes in the invasive tumors, indicating broad transcriptional differences between invasive and non-invasive NFPAs. Unsupervised hierarchical clustering of the top 50 DEGs separated invasive from non-invasive sample groups (Figure 2A). visualized the genome-wide distribution of DEGs, with the five subsequently validated core genes highlighted (Figure 2B).

To identify co-expression modules associated with invasiveness, we next performed weighted gene co-expression network analysis (WGCNA), which grouped genes into nine modules (Figure 2C). Module–trait correlation analysis showed that the turquoise module was most strongly associated with the invasive phenotype (Pearson r = 0.99, *p* = 3 × 10^−28^) and contained 3655 genes; therefore, it was selected as the core invasiveness-associated module for downstream analysis (Figure 2D).

### 3.2. Identification of a Core Invasiveness Gene Signature

**Figure 3:**
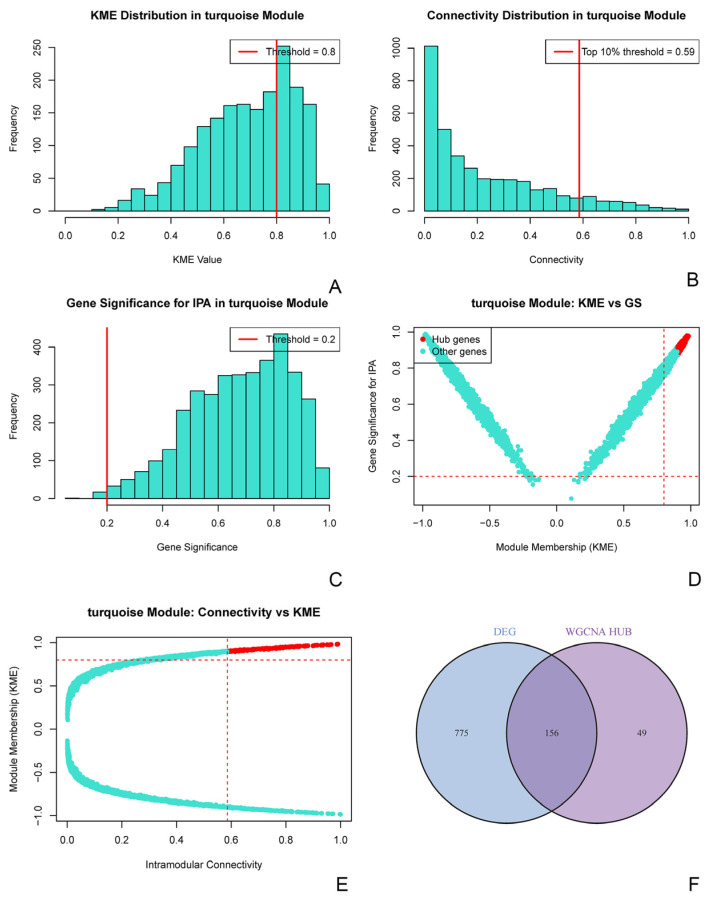
Stepwise screening of hub genes yields a high-confidence candidate gene set from the invasiveness-associated module. (**A**–**C**) Sequential filtering criteria applied within the turquoise module: (**A**) Genes with high module membership (KME ≥ 0.8). (**B**) Genes in the top 10% of intramodular connectivity (kWithin). (**C**) Genes significantly associated with invasiveness (Gene Significance, GS ≥ 0.2). (**D**) Scatter plot of module membership (KME) versus gene significance (GS) for invasiveness. Hub genes (red) are concentrated in the high-KME and high-GS region, validating the screening criteria. (**E**) Scatter plot of intramodular connectivity versus module membership (KME), demonstrating that the identified hub genes (red) exhibit both high connectivity and high module membership. (**F**) A Venn diagram between the 205 hub genes and the 931 differentially expressed genes (DEGs) yields the final 156 high-confidence candidate genes for downstream validation.

To identify key regulatory genes within the core invasiveness-associated module, we applied a stepwise screening strategy. Within the turquoise module, stringent filtering based on intramodular connectivity, module membership, and association with invasiveness yielded 205 hub genes. Intersecting these hub genes with the previously identified DEGs yielded a high-confidence candidate set of 156 genes. DEGs produced a high-confidence candidate set of 156 genes with both network centrality and differential expression (Figure 3).

**Figure 4:**
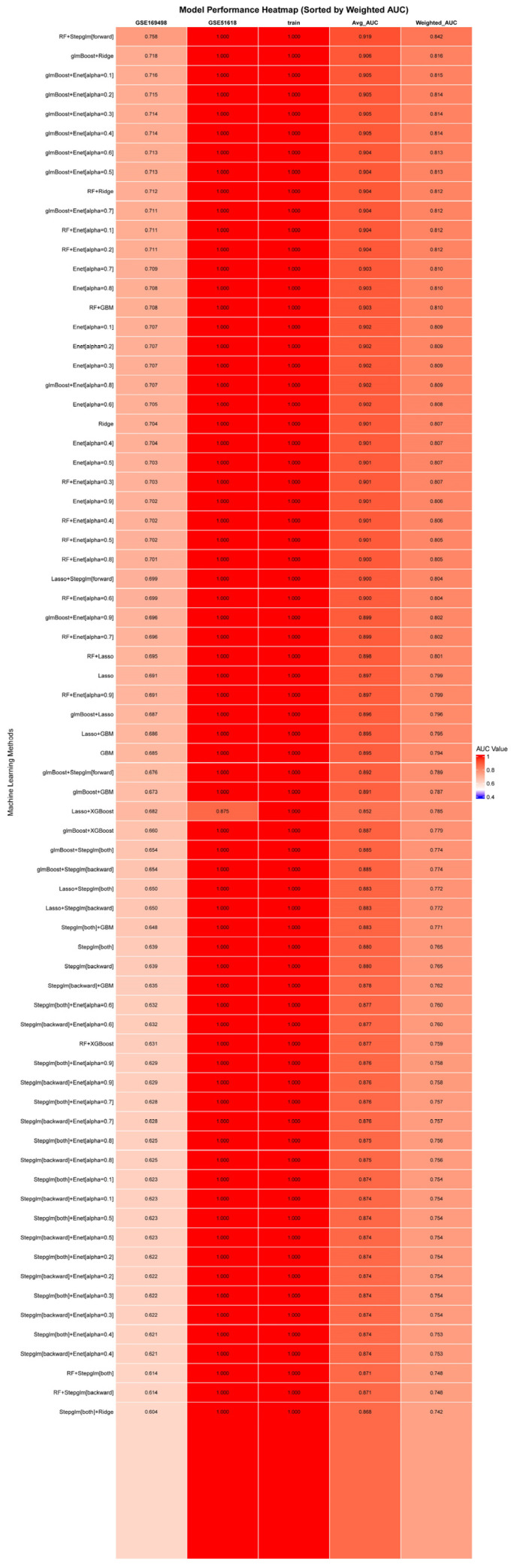
Identification of the optimal predictive model and key signature genes. The RF + Stepglm[forward] model demonstrated the best performance (mean AUC = 0.919; sample-size-weighted AUC = 0.842) and was used to select 71 key signature genes for subsequent analysis.

To evaluate the diagnostic utility of these candidates, we conducted cross-dataset machine learning validation using two independent public transcriptomic datasets (GSE169498, *n* = 73; GSE51618, *n* = 7). Among 113 evaluated algorithms, the Random Forest combined with forward stepwise logistic regression (RF + Stepglm[forward]) model achieved the best discriminative (mean AUC = 0.919) and was used to select 71 feature genes for downstream analyses (Figure 4).

### 3.3. Functional Enrichment and Coordinated Biological Implications of the Core Gene Signature

**Figure 5:**
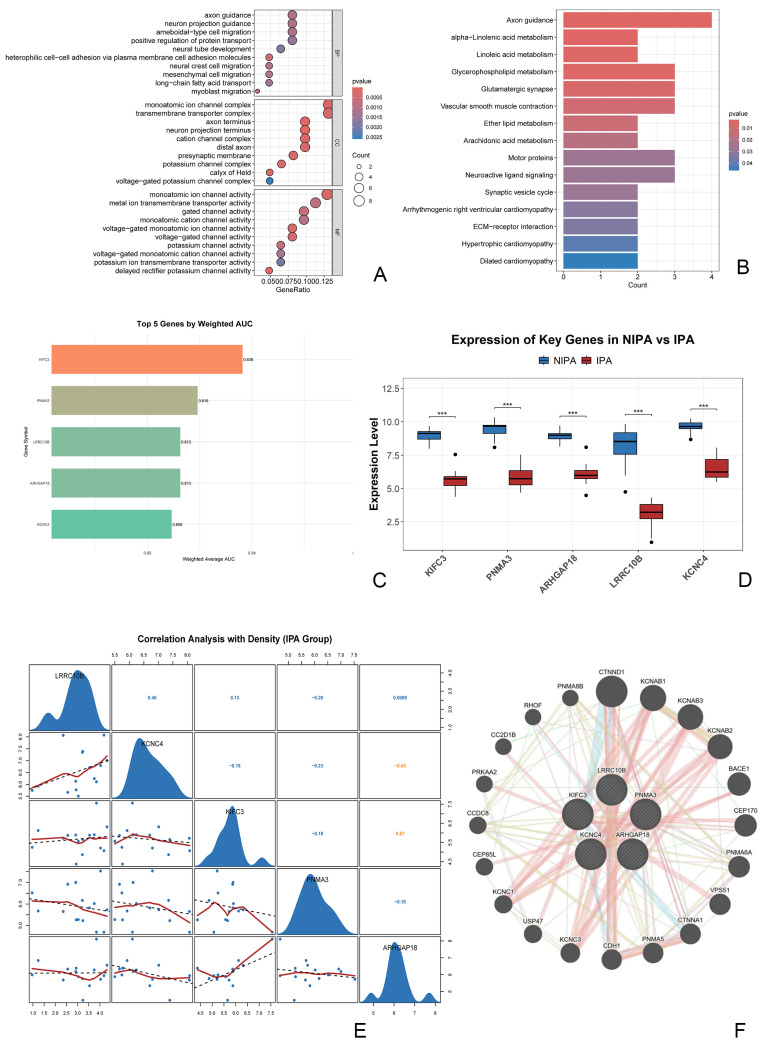
Functional enrichment, machine learning prioritization, and interaction network of the core signature genes. (**A**) Gene Ontology (GO) enrichment analysis of the 71 key feature genes, showing the top 20 significantly enriched terms related to axon guidance, neuron projection, and transmembrane transporter complex. (**B**) Kyoto Encyclopedia of Genes and Genomes (KEGG) pathway enrichment, highlighting significant associations with Axon guidance, Neuroactive ligand-receptor interaction, and Oxidative phosphorylation. (**C**) The five core signature genes ranked by their weighted diagnostic AUC values across validation cohorts, with KIFC3 exhibiting the highest performance. (**D**) Expression validation in the internal cohort confirms significant downregulation of all five genes in invasive NFPAs compared to non-invasive tumors (*** *p* < 0.001). (**E**) Pairwise correlation analysis among the five genes reveals a significant positive correlation between KIFC3 and ARHGAP18 (Pearson r = 0.57) (Blue indicates weak or no correlation (|r| ≤ 0.4), orange indicates moderate to strong correlation (|r| > 0.4)). (**F**) Protein-protein interaction network of the five core genes generated by GeneMANIA. The network, supported primarily by evidence of physical interactions, shows that the signature genes form a cohesive module enriched in functions related to ion channel complexes and transmembrane transport.

To clarify the biological relevance of the core gene signature, we performed functional enrichment analyses of the machine learning–prioritized genes. Gene Ontology (GO) enrichment indicated overrepresentation of terms related to transmembrane transporter complexes, monoatomic ion channel complexes, and axon guidance (Figure 5A). Consistently, Kyoto Encyclopedia of Genes and Genomes (KEGG) analysis highlighted pathways including axon guidance and neuroactive ligand-receptor interaction (Figure 5B).

Univariate diagnostic evaluation of the 71 feature genes further showed that KIFC3, PNMA3, ARHGAP18, LRRC10B, and KCNC4 achieved the highest AUC values; therefore, these genes were defined as the five-gene signature for invasive nonfunctioning pituitary adenomas (Figure 5C). In the internal cohort, all five genes were significantly downregulated in invasive tumors compared with non-invasive tumors (all *p*-values < 0.01; Figure 5D). In addition, expression correlation analysis identified a significant positive association between KIFC3 and ARHGAP18 (Pearson r = 0.57), suggesting potential coordinated regulation (Figure 5E). Finally, a GeneMANIA-derived interaction network indicated that these five genes form a connected functional module, with associated genes enriched for terms such as potassium channel complex and transmembrane ion transport (Figure 5F).

**Figure 6:**
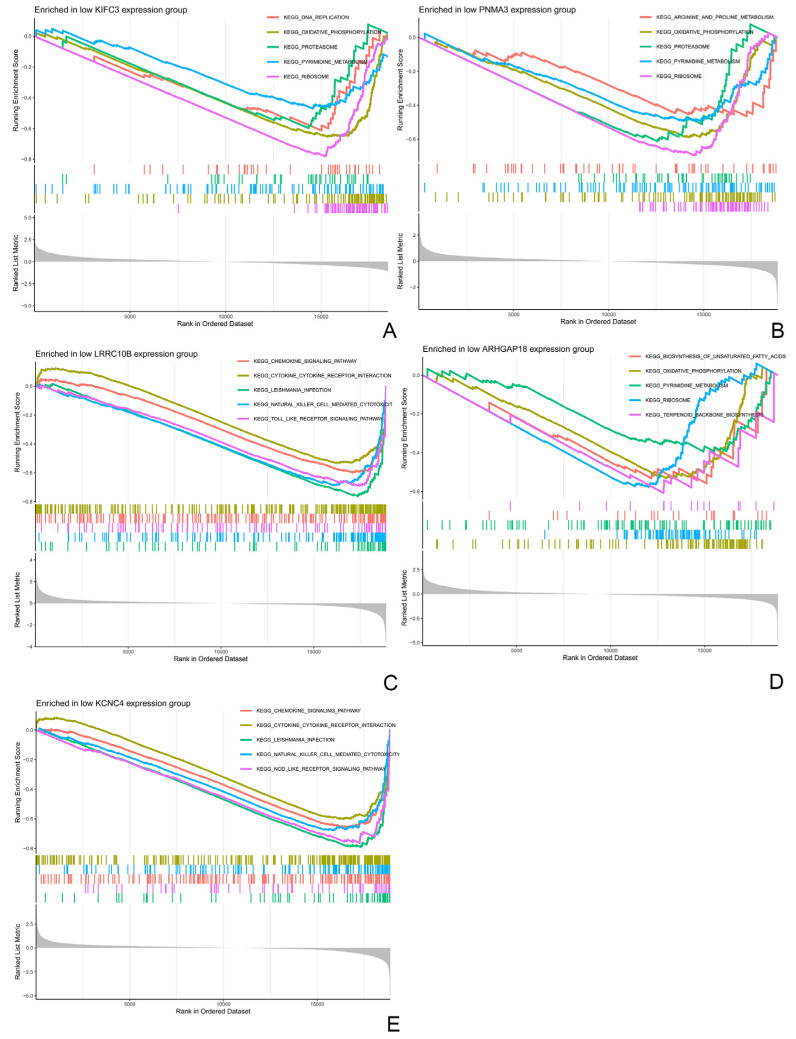
Gene set enrichment analysis (GSEA) of the core signature genes. GSEA was performed based on the expression pattern of each core gene. Plots (**A**–**E**) correspond to KIFC3, PNMA3, ARHGAP18, LRRC10B, and KCNC4, respectively. The top five significantly enriched KEGG pathways (FDR < 0.25) for each gene are listed. The coordinated downregulation of these five genes is associated with the suppression of pathways, including oxidative phosphorylation and neuroactive ligand-receptor interaction in invasive NFPAs.

Gene Set Enrichment Analysis (GSEA) further suggested that coordinated downregulation of this core gene set was associated with reduced enrichment of pathways, including oxidative phosphorylation and neuroactive ligand-receptor interaction in invasive samples (Figure 6). Taken together, these results link the signature to alterations in energy metabolism–related and neuroactive signaling pathways in invasive nonfunctioning pituitary adenomas.

### 3.4. DNA Methylation Association Analysis of Core Genes in an Independent Cohort

To examine whether transcriptional downregulation of core genes is associated with epigenetic regulation, we analyzed public DNA methylation datasets. Discovery analyses were performed in an Illumina EPIC 850K cohort (GSE207937, *n* = 44), and reproducibility was assessed in an Illumina 450K cohort (GSE115783, *n* = 34). Given differences in array design and probe coverage, the cohorts were analyzed separately rather than merged to enable an objective cross-platform assessment of robustness.

**Figure 7:**
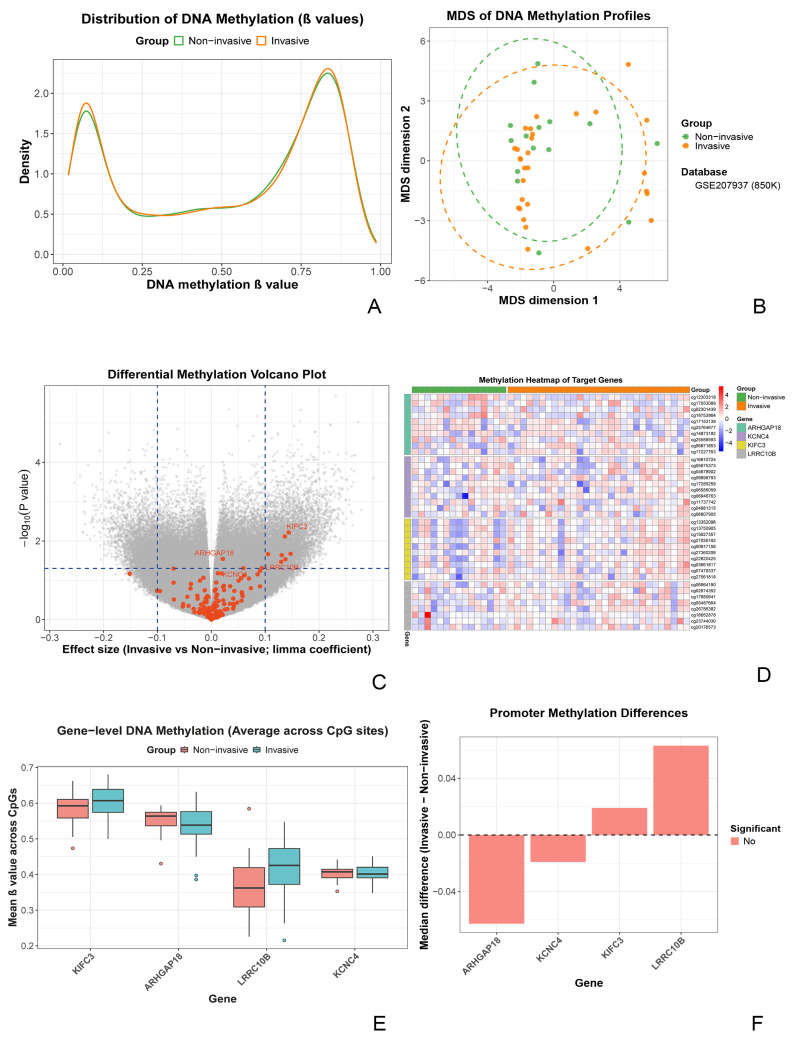
DNA methylation analysis of core genes in invasive versus non-invasive non-functioning pituitary adenomas. (**A**) Density plot of genome-wide methylation beta values, showing a high degree of overlap in the overall methylation profiles between the invasive and non-invasive groups. (**B**) Multidimensional scaling (MDS) plot based on genome-wide methylation data, reflecting the similarity of global methylation patterns among samples with no clear separation between the two groups. (**C**) Volcano plot of differential methylation analysis. Each point represents an individual CpG site. Several sites associated with core genes (e.g., within KIFC3 and ARHGAP18) met the threshold for differential methylation (|Δβ| > 0.1 and *p* < 0.05, indicated by dotted lines). (**D**) Heatmap displaying the methylation levels of all analyzed CpG sites associated with the core genes across individual samples from both groups. (**E**) Comparison of the average methylation levels across all CpG sites associated with each core gene locus. No statistically significant differences were observed between groups for any gene. (**F**) Comparison of the average methylation levels specifically within the promoter regions of the core genes. No genes showed significant promoter methylation differences between groups. (Note: Analysis for PNMA3 is not included in panels due to insufficient probe coverage on the array platforms.)

In the discovery cohort (GSE207937), β-value density distributions overlapped substantially between invasive and non-invasive groups, indicating broadly similar global methylation profiles (Figure 7A). Likewise, multidimensional scaling (MDS) did not reveal a clear separation between groups at the genome-wide level (Figure 7B). Using a commonly applied threshold for differentially methylated positions (DMPs) (nominal *p* < 0.05 and |Δβ| > 0.10), volcano plot visualization highlighted several CpG sites annotated to the core genes (e.g., within KIFC3 and ARHGAP18) that met these criteria (Figure 7C).

To determine whether these locus-specific signals reflected broader methylation changes, we compared mean methylation levels across all CpG sites mapped to each core gene. No statistically significant differences were observed between invasive and non-invasive groups for any gene (Figure 7E). Notably, PNMA3 was excluded from this analysis due to insufficient probe coverage on both the 850K and 450K array platforms. Subsequently, promoter region analysis (TSS ± 1500 bp) was performed for the four remaining genes with adequate probe coverage, which also revealed no significant methylation differences between groups (Figure 7F). Collectively, these results suggest that the observed CpG-level signals are likely localized to specific regulatory regions rather than reflecting widespread methylation alterations across gene bodies or promoter regions.

In the validation cohort (GSE115783), promoter methylation of ARHGAP18 showed a nominal increase in the invasive tumors (*p* = 0.0425); however, the effect size was small, and this difference was not reproduced in the discovery analysis (Figure S2). Overall, although several CpG sites near core genes met the DMP criteria in volcano plot analysis, gene-level and promoter-level methylation assessments did not identify consistent, reproducible differences across datasets. These results suggest that reduced expression of the core signature in invasive nonfunctioning pituitary adenomas is unlikely to be independently explained by DNA methylation changes at the gene or promoter level and may instead involve other regulatory mechanisms (e.g., histone modifications, transcriptional regulation, or distal regulatory elements not captured by promoter-focused probes).

## 4. Discussion

This study employed an integrative multi-omics framework combining weighted gene co-expression network analysis (WGCNA), machine learning validation, and epigenetic profiling to identify potential molecular signatures associated with NFPA invasiveness. Through this approach, we observed coordinated downregulation of a five-gene signature (KIFC3, PNMA3, ARHGAP18, LRRC10B, and KCNC4) in invasive NFPAs compared to non-invasive tumors.

This work integrates network-based analysis, machine learning validation, and epigenetic profiling through three complementary analytical layers: (1) unbiased network construction using WGCNA to identify co-regulated gene modules rather than relying solely on differential expression; (2) machine learning–based feature selection (Random Forest combined with forward stepwise logistic regression) to prioritize biomarkers with cross-dataset generalizability (mean AUC = 0.919); and (3) independent epigenetic validation using public DNA methylation datasets.

Together, this pipeline moves beyond single-gene correlation analyses toward a systems-level characterization of molecular patterns associated with invasiveness in nonfunctioning pituitary adenomas. We propose that such integrative approaches can help prioritize candidates for downstream functional validation, particularly for heterogeneous tumors in which single-omic analyses may capture only part of the molecular landscape.

### 4.1. Putative Mechanisms: Functional Grouping of the Signature Genes

The coordinated downregulation of the five signature genes in invasive nonfunctioning pituitary adenomas contrasts with reported pro-tumorigenic roles of these genes in several malignant settings. For interpretability, these genes can be broadly grouped into two functional categories.

The first category relates to cytoskeletal dynamics and cell-cycle-associated processes. KIFC3, a kinesin superfamily motor protein, contributes to intracellular cargo transport and mitotic spindle organization [11]. In several cancers, KIFC3 overexpression has been linked to increased proliferation and invasion, potentially through PI3K/AKT/mTOR signaling [10,12]. In contrast, we observed KIFC3 downregulation in invasive nonfunctioning pituitary adenomas.

Similarly, ARHGAP18 encodes a Rho GTPase-activating protein involved in cytoskeletal remodeling and cell migration through RhoA-associated signaling. exhibiting context-dependent dual roles—suppressing gastric cancer progression via MAPK pathway inhibition while paradoxically promoting hepatocellular carcinoma proliferation [13,14]. The downregulation of both genes in invasive NFPAs suggests potential disruption of cytoskeletal integrity.

The second category relates to ion channel activity and neuroendocrine signaling. KCNC4 encodes the Kv3.4 voltage-gated potassium channel, which promotes cancer cell migration via vimentin regulation and AKT pathway activation, as demonstrated in head and neck squamous cell carcinoma, osteosarcoma, and cervical cancer [15,16,17]. PNMA3, a paraneoplastic antigen family member, shows restricted expression in neural and testicular tissues with limited functional characterization in tumorigenesis [18]. LRRC10B, a leucine-rich repeat protein, has been implicated in cell proliferation and adhesion in lung adenocarcinoma and clear cell renal cell carcinoma [19].

The coordinated downregulation of these functionally diverse genes in invasive nonfunctioning pituitary adenomas may be consistent with alterations in neuroactive ligand-receptor interaction and oxidative phosphorylation pathways, potentially affecting local microenvironmental signaling and energy metabolism. However, these interpretations remain speculative, and the observed expression patterns may represent downstream correlates rather than direct drivers of invasiveness; mechanistic validation will be required.

### 4.2. Comparison with Previous Studies and Methodological Novelty

In prior work on invasiveness in nonfunctioning pituitary adenomas, Cheng et al. (2019) reported that KIFC3 downregulation by increased methylation may be associated with greater invasiveness [20]. However, that study did not construct a functional module of interrelated genes. In our re-analysis of their publicly available dataset, ARHGAP18, LRRC10B, and KCNC4 exhibited similar methylation patterns, although these genes were not emphasized as candidate markers in the original report.

### 4.3. Limitations

This discrepancy highlights methodological differences between studies. Cheng et al. restricted their analysis to specific NFPA subtypes (gonadotroph and null cell adenomas) and primarily used pairwise correlation analyses. In contrast, our framework integrates WGCNA-based module discovery with machine learning validation across three independent cohorts, allowing identification of genes with high intramodular connectivity that may not exhibit extreme differential expression. Subsequent machine learning validation prioritized robust biomarkers with cross-dataset generalizability.

The observed differences in absolute methylation levels between our study and Cheng et al. may reflect technical variations (e.g., different Illumina array platforms, batch effects) or biological factors such as tumor heterogeneity and differences in cohort composition. Although both studies suggest a trend toward hypermethylation accompanying downregulation, the modest effect sizes observed in our analysis suggest that DNA methylation likely represents one of multiple regulatory layers rather than the sole determinant of expression changes.

Several limitations should be acknowledged. First, the sample sizes remain modest (internal cohort: *n* = 32; validation cohorts combined: *n* = 80), which may limit statistical power and generalizability; our post-hoc sensitivity analysis suggests adequate power primarily for large effect sizes (Cohen’s d > 1.0). Second, the present analyses are associative rather than causal. For example, correlations between CpG methylation changes near KIFC3 and reduced expression do not demonstrate direct methylation-driven silencing, and other regulatory mechanisms (e.g., transcription factor binding and chromatin remodeling) may contribute. Third, the methylation and transcriptomic analyses were performed in independent cohorts without matched multi-omic profiling, precluding definitive assessment of the methylation-expression relationship within identical samples. Finally, functional perturbation experiments (e.g., CRISPR-mediated gene editing, methylation editing) will be required to establish causal roles in NFPA invasion.

### 4.4. Future Directions and Clinical Implications

Pending experimental validation, the five-gene signature may support preoperative risk stratification when combined with conventional imaging and histopathological assessment. Locus-specific methylation signals raise the possibility of targeted epigenetic modulation; however, any therapeutic implications remain speculative at this stage. Future work should prioritize: (1) prospective multicenter validation in larger cohorts; (2) functional studies to establish causal roles of the signature genes in NFPA invasion using in vitro and in vivo models; (3) matched multi-omic profiling to clarify methylation–expression relationships within the same tumors; and (4) evaluation of whether restoring signature gene expression (e.g., via epigenetic modulators) attenuates invasive phenotypes in preclinical models.

In summary, this exploratory study presents an integrative multi-omics framework for identifying candidate biomarkers of invasiveness in nonfunctioning pituitary adenomas. Although the five-gene signature and the associated methylation patterns require rigorous functional validation, they provide testable hypotheses for understanding molecular mechanisms underlying aggressive tumor behavior.

## 5. Conclusions

This study identified a robust five-gene invasiveness signature (KIFC3, PNMA3, ARHGAP18, LRRC10B, KCNC4) in NFPAs through an integrated multi-omics pipeline. The coordinated downregulation of this signature in invasive tumors was associated with altered enrichment of pathways related to oxidative phosphorylation and neuroactive ligand–receptor interaction. Machine learning validation across independent cohorts confirmed the strong diagnostic potential of this signature, with the RF + Stepglm[forward] model achieving an AUC of 0.919. Although initial screening suggested potential locus-specific DNA methylation variations near certain core genes, subsequent rigorous analyses at the gene-wide and promoter-wide levels did not identify robust or widespread methylation differences associated with their downregulation. This suggests that transcriptional suppression of these genes is more likely driven by alternative regulatory mechanisms rather than being independently driven by DNA methylation. Collectively, these findings present a novel molecular signature with potential utility for stratifying NFPA invasiveness. They also underscore the complexity of the regulatory landscape in aggressive tumors and highlight the necessity for future functional studies and integrative multi-omics approaches to fully elucidate the upstream mechanisms driving this invasive phenotype.

## Data Availability

The original contributions presented in this study are included in the article/Supplementary Material. Further inquiries can be directed to the corresponding authors.

## Supplementary Materials

The following supporting information can be downloaded at: https://www.mdpi.com/article/10.3390/biomedicines14020484/s1, Figure S1: Post-hoc sensitivity analysis of the internal cohort; Figure S2: Validation of DNA methylation patterns in the independent cohort GSE115783 (450K array, *n* = 34).

## Abbreviations

The following abbreviations are used in this manuscript:

NFPAs	Non-functional pituitary adenomas
WGCNA	Weighted gene co-expression network analysis
FPAs	Functional pituitary adenomas
DEGs	Differentially expressed genes
GO	Gene Ontology
KEGG	Kyoto Encyclopedia of Genes and Genomes
BMIQ	Beta-Mixture Quantile Dilation
DMPs	Differentially methylated positions
