# Supplementary material for: Identification and Validation of Signature Genes in Invasiveness-Associated Modules of Nonfunctioning Pituitary Adenomas
Source: Biomedicines. 2026 Feb 23;14(2):484. doi: 10.3390/biomedicines14020484 (PMC12938159; doi:10.3390/biomedicines14020484)
Supplement: Supplementary file 1 [file biomedicines-14-00484-s001.zip › biomedicines-4113281-supplementary.pdf]

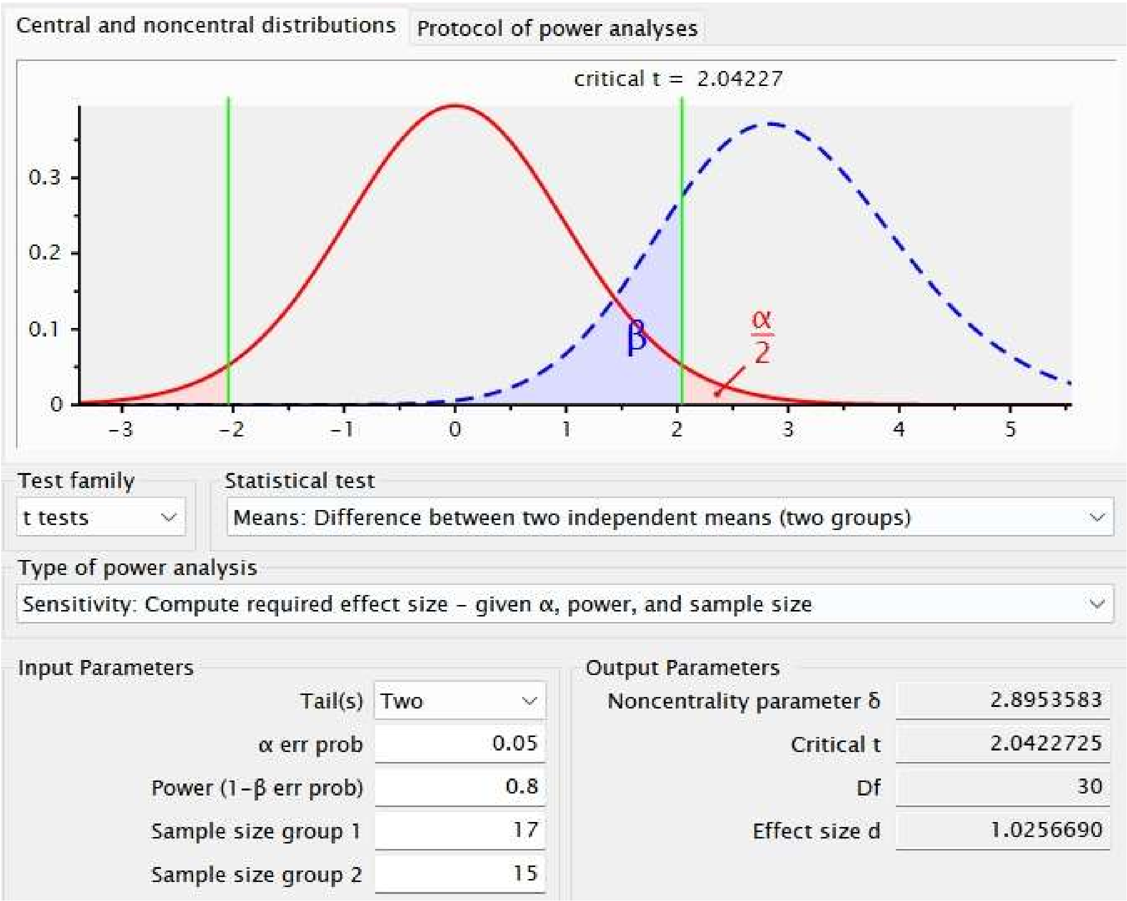

Figure S1. Post-hoc sensitivity analysis of the internal cohort. The analysis was performed using G*Power (version 3.1.9.7) to determine the minimum detectable effect size given the fixed sample size. Parameters were set for a two-sided, two-sample t-test with a Type I error rate (α) of 0.05 and statistical power (1-β) of 0.80. Inputting the actual sample sizes of the non-invasive (n=17) and invasive (n=15) groups yielded a minimum detectable standardized effect size (Cohen's d) of 1.03.

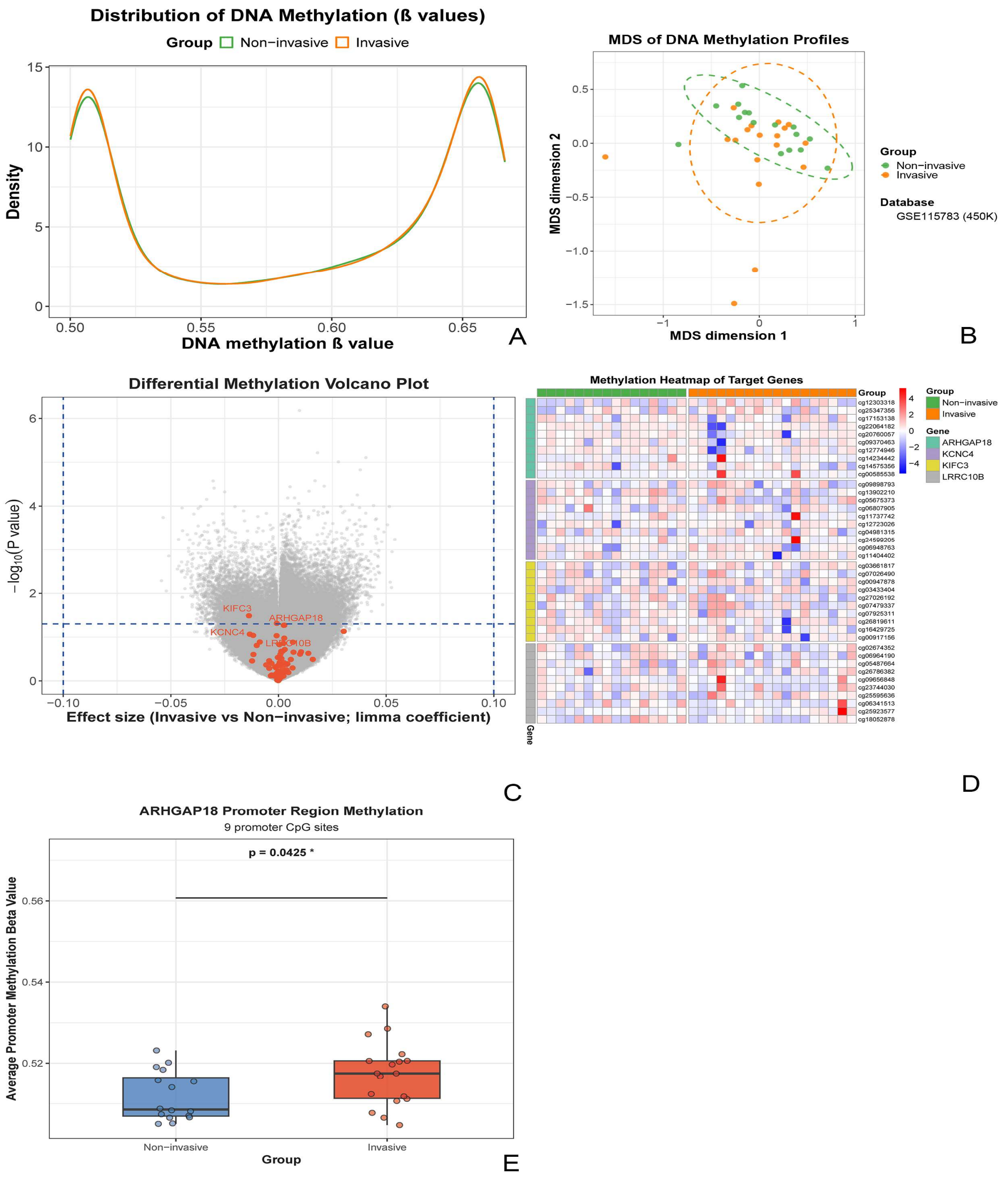

Figure S2. Validation of DNA methylation patterns in the independent cohort GSE115783 (450K array, n=34). (A) Density plot of genome-wide methylation β-values showing substantial overlap between invasive and non-invasive groups. (B) Multidimensional scaling (MDS) plot based on genome-wide methylation data, with dashed ellipses indicating partial separation between groups. (C) Volcano plot of differential methylation analysis. Several CpG sites associated with core signature genes (KIFC3, ARHGAP18, KCNC4, LRRC10B) are highlighted in red. (D) Heatmap displaying methylation levels of CpG sites associated with the core genes across individual samples. (E) Box plot comparing average promoter methylation levels of ARHGAP18 between groups. The invasive group showed nominally higher methylation ($p = 0.0425$), though this difference was not reproduced in the primary analysis cohort (GSE207937). PNMA3 was excluded from analysis due to insufficient probe coverage on the 450K platform.
